# 4 Day in dry immersion reproduces partially the aging effect on the arteries as observed during 6 month spaceflight or confinement

**DOI:** 10.1038/s41526-021-00172-6

**Published:** 2021-11-02

**Authors:** Danielle Greaves, Laurent Guillon, Stephane Besnard, Nastassia Navasiolava, Philippe Arbeille

**Affiliations:** 1 UMPS-CERCOM (Unite Medecine Physiologie spatiale) Faculte de Medecine, Tours, France; 2 Université de Normandie, UFR Sante, Caen, France; 3 Centre de Recherche Clinique du CHU d’Angers, Tours, France

**Keywords:** Health care, Risk factors

## Abstract

The objectives of this study were to determine whether 4 days of dry immersion (DI) induced similar arterial aging as spaceflight and to test the impact of thigh cuffs. Eighteen subjects underwent DI; nine wore thigh cuffs. Cardiac and arterial targets were assessed by ultrasound. No significant differences were found between the groups. The left ventricle volume, stroke volume (SV), and ejection fraction decreased with DI (*p* < 0.001). Carotid distensibility reduced (*p* < 0.05), carotid to femoral arterial tree became stiffer in 33% of the subjects, and femoral artery intima media thickness increased (*p* < 0.05). A reduction in plasma volume is likely to have caused the observed cardiac changes, whereas the arterial wall changes are probably best explained by hypokinesia and/or environmental stress. These changes are similar but lower in amplitude than those observed in spaceflight and mimic the natural aging effect on earth. The daytime-worn thigh cuffs had no acute or chronic impact on these arterial-focused measurements.

## Introduction

Several years ago, significant cardiovascular changes were reported during spaceflights and head-down bed rests (HDBRs), such as decreases in the left ventricle diastolic volume (LVDV), stroke volume (SV), decreases in limb arterial vascular resistance, or alteration of the orthostatic tolerance, right after these experiments^1–3^. Such changes occurred within the first day of HDBR or spaceflight, and were related to the abrupt cephalad fluid transfer induced by the environment (microgravity) or the position (head-down position), and the subsequent adaptation. Later on, cardiac mass decrease was measured, which corresponded to a cellular adaptation to the new environmental condition, which included hypovolemia and the absence of exercise^4,5^.

More recently, carotid and femoral intima media thickness (IMT) were found to be increased and the wall distensibility decreased in long-duration confinement (1 G) and long-duration spaceflight (0 G)^6^. The amplitude of these changes (15–20%) were similar to those induced by 20 years of aging in a normal population. The ground-based confinement results, in particular, could not be explained by fluid transfer (as in spaceflight) and subsequent flow and pressure redistribution. Moreover, they could not be explained exclusively by reduction in physical activity nor abnormal nutritional regime; exercise and nutrition are supposedly well-controlled in spaceflight and confinement studies. On the other hand, a significant increase in carotid artery wall stiffness was found alongside with disturbances in glucose metabolism^7^. These results suggest that several factors acting together could be at least partially responsible for the accelerated aging of the arterial wall including inflammatory processes, insufficient physical activity, and environmental and psychological stresses.

Dry immersion (DI) has been proposed to simulate the fluidshift-related adaptations of the cardiac arterial and venous system. DI induces a sustained and prolonged pressure over the body from the neck down to the feet, causing a fluid transfer in the cephalad direction sustained over several days^2,8^. In a previous 3-day DI study, the measures on the first day reported a significant increase in jugular and portal vein volume, and in cerebral vein velocity^2^, which confirmed that DI induces similar changes in the venous system similar to that observed in spaceflight and HDBR at least during the first day. In addition to the induced fluidshift towards the head, DI keep the participant practically motionless. The body rests in the same semi-recumbent position for 24 h/day, which may generates stress, lower back pain, and uncomfortable sleep during several days. According to our findings in spaceflight at the cardiac and arterial level, we hypothesized that the total absence of physical movement, the fluidshift, and the stress environment may induce modification of the cardiac and arterial walls. Dry immersion provides a potentially stronger stimulus than HDBR for certain physiological systems such as cardiovascular and muscle. Dry immersion provides an alternative, complimentary analog method, in addition to HDBR, for investigating hypotheses prior to manifest on a flight mission and countermeasures.

On the other hand, simple immersion in water creates the same effect but for long immersions (e.g., several hours/days), the “dry” part of dry immersion is more acceptable to participants due to skin irritation from longer exposure with water.

As we do not know the role of flow and pressure redistribution (fluidshift-induced) on the modification of the cardiovascular structures inflight, we tested the thigh cuffs countermeasure most commonly used to attenuate the fluidshift with the objective to see whether it has an impact on the expected cardiac and arterial changes. Thigh cuffs (Braselets) were designed to stow a significant amount of fluids into the legs and subsequently reduce the fluidshift toward the upper part of the body. As the dry immersion mimics the headwards fluidshift and maintain the subject motionless and without physical activity, it was suggested to evaluate the effects of thigh cuffs on most of the physiological systems affected by dry immersion (cardiovascular, muscle, bone and glucose metabolism, etc.). Other well-known method to counteract the fluidshift effect (i.e., Lower body negative pressure (LBNP)) and physical inactivity consequences such as orthostatic intolerance, muscle tone, and vein compliance decrease (foot stimulation) were successfully tested^9,10^.

Thigh cuffs, when used in space or HDBR, have been found to reduce the fluidshift towards the cervical area and change the cardiac stroke volume and the flow redistribution^11,12^. Thus, we compared our results in dry immersion with these previous findings and hypothesized some similarities (if any) in the effects of the countermeasure and the existence or absence of memory effect from one day to the next.

In the present 5-day DI study, our objective was to detect and measure any changes in cardiac volume, carotid, femoral and tibial wall properties, regional flow redistribution, and vascular resistance, and compare with cardiac and arterial inflight (International Space Station (ISS)) or with confinement changes. Secondarily, to evaluate and quantify the efficiency of thigh cuffs when used during daytime only, similar to what is currently done in the Russian segments on ISS.

## Results

Participant characteristics are listed in Table 1. The first hypothesis was to test the chronic effect of the dry immersion with and without daytime thigh cuffs. This was achieved by testing baseline (Pre) against the fourth day, in the morning (D4 AM) with main effects of time (DI effect) and condition (with or without thigh cuff). Four days in dry immersion changed significantly several cardiac and arterial parameters in both control and thigh-cuff subjects but there was no significant difference between these two groups. This means that there was no difference if the cuffs were worn for the previous three daytime periods or not. Each graph indicates individual data, with cuff and control groups divided for visualization. Results are reported as pooled for all 18 participants.

**Table 1:** Baseline group characteristics at DI-2, mean ± SD.

	Age (years)	Height at selection (cm)	DI-2 weight (kg)	DI-2 BMI (kg/m^2^)	DI-2 VO_2_max (ml/min/kg)	DI-2 morning HR (b.p.m.)	DI-2 morn *T* (°C)	DI-2 morning SBP (mm Hg)	DI-2 morning DBP (mm Hg)
Control (*n* = 9)	33.9 ± 7.1	176 ± 6	73.9 ± 7.5	23.9 ± 1.7	46.5 ± 8.1	57 ± 6	36.4 ± 0.3	115 ± 11	68 ± 5
Cuffs (*n* = 9)	34.1 ± 3.7	180 ± 4	74.3 ± 8.8	22.7 ± 1.8	46.9 ± 5.8	58 ± 8	36.4 ± 0.5	117 ± 10	68 ± 9

Unpaired *T*-test did not reveal significant differences between groups.

### Cardiac structure and function (Fig. 1)

At D4 AM, the left ventricular diastolic volume was significantly lowered from 164.6 ± 42.9 ml to 152.5 ± 37.2 ml (mean −8% loss, *p* = 0.0293), whereas the left ventricular systolic volume was unchanged (*p* > 0.05). Stroke volume was significantly reduced (108.4 ± 27.5 ml pre to 93.3 ± 22.7 ml at D4 AM; mean −14%, *p* < 0.001) and also the ejection fraction (0.66 ± 0.06% pre to 0.62 ± 0.07% at D4 AM, mean −6%, *p* < 0,01). Cardiac mass showed a tendency to decrease (200 ± 60 g pre to 180 ± 40 g at D4, mean −7%, *p* = 0.0613—on 11/18 subjects).

**Fig. 1:**
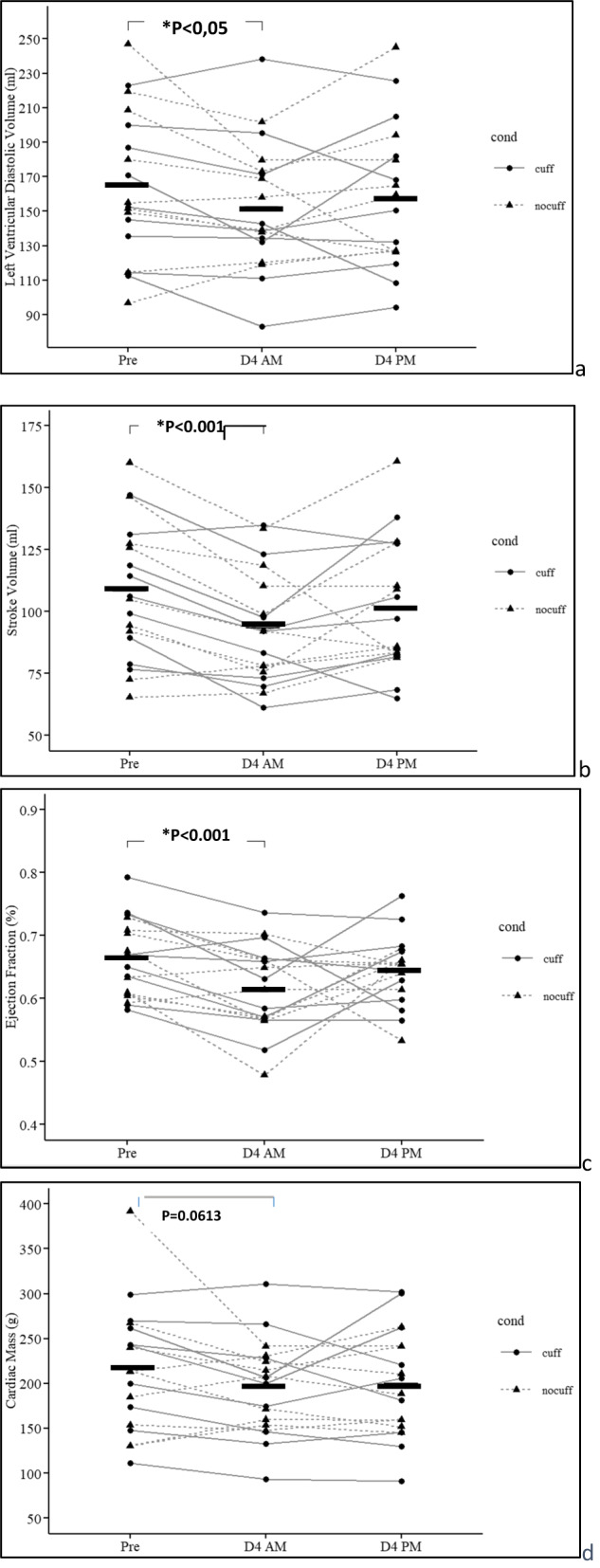
Cardiac function outcomes from 4 days of dry immersion. Bold horizontal bars represent the mean. DI effect from Pre to D4 AM on the 2 gr averaged (*p) is reported as “*p < 0.0*x*” and is plotted on top of the diagram. The change from D4 AM to D4 PM (with or without cuffs) are not significant for any parameter (*p* not reported). Pre was prior to dry immersion; D4 AM represents 4 days of dry immersion (morning prior to donning cuffs for the day for the cuff gr). D4 PM represent the data in the afternoon of day 4, after 10 h with cuff on, for the cuff gr.

### Arterial wall properties (Fig. 2)

Carotid artery distensibility index dropped significantly from 0.108 ± 0.024 units to 0.097 ± 0.026 units (mean −9%, *p* = 0.041), whereas the carotid-femoral pulse wave velocity and carotid to tibial pulse wave velocity tended to decrease in 11/18 subjects (*p* = 0.07).

**Fig. 2:**
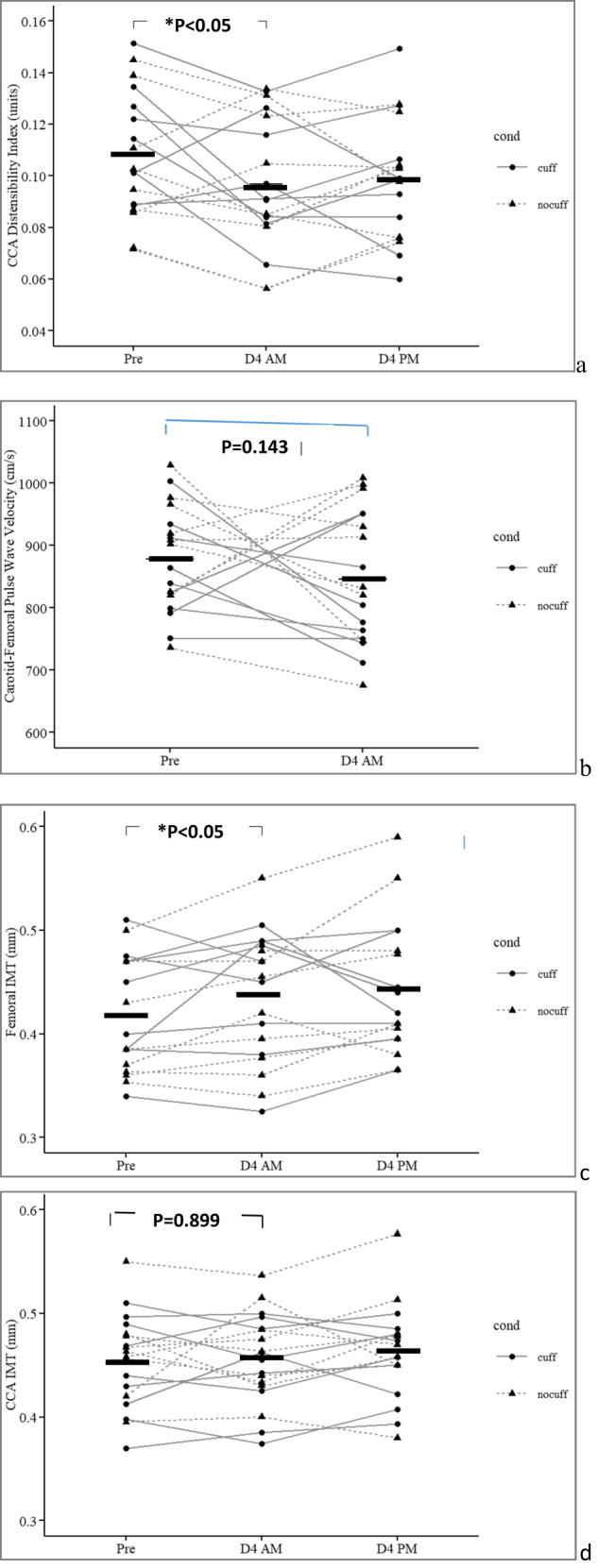
Arterial wall properties after 4 days of dry immersion. Horizontal black bars represent the mean. DI effect from Pre to D4 AM on the 2 gr averaged (*p) is reported as “*p < 0.0*x*” and plotted on top of the diagram. The change from D4 AM to D4 PM (with or without cuffs) are not significant for any parameter (*p* not reported). Pre was prior to dry immersion; D4 AM represents 4 days of dry immersion (morning prior to donning cuffs for the day for the cuff gr). D4 PM represent the data in the afternoon of day 4, after 10 h with cuff on, for the cuff gr.

The IMT of the superficial femoral artery was, on average, 4% thicker (0.42 ± 0.056 to 0.44 ± 0.063 mm; *p* < 0.05), whereas the carotid IMT did not show significant change (Fig. 2).

### Arterial hemodynamics (Fig. 3)

**Fig. 3:**
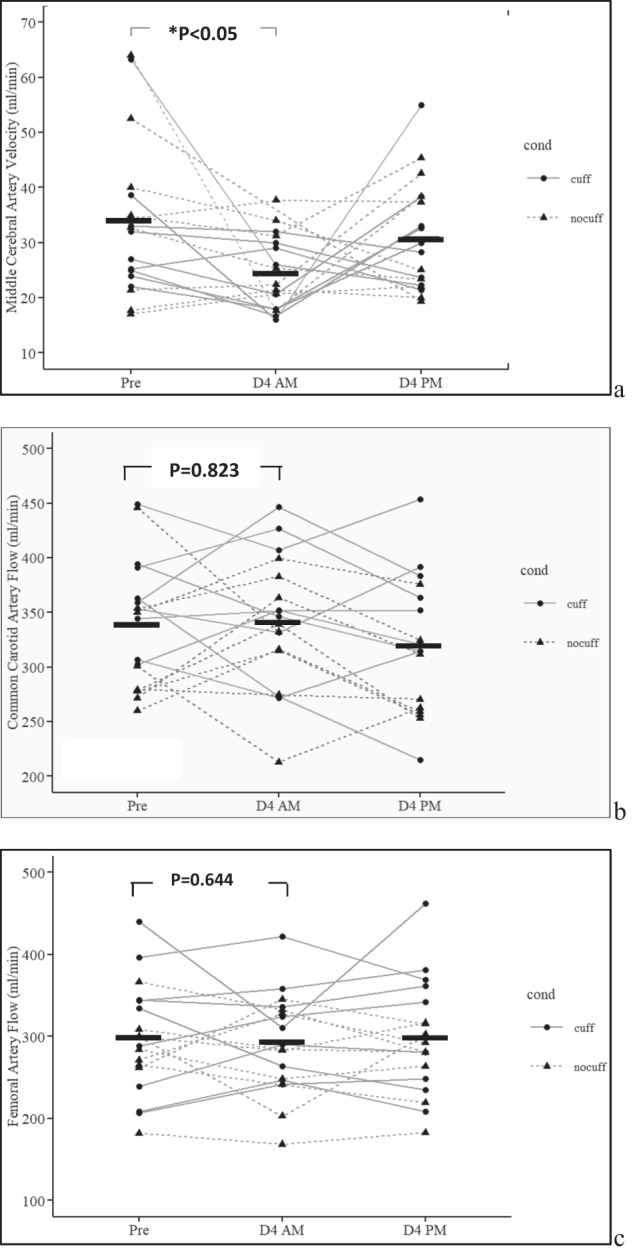
Cerebral, carotid, and femoral flow velocity after 4 days of dry immersion. Horizontal black bars represent the mean. DI effect from Pre to D4 AM on the 2 gr averaged (*p) is reported as “*p < 0.0*x*” and plotted on top of the diagram. The change from D4 AM to D4 PM (with or without cuffs) are not significant for any parameter (*p* not reported). Pre was prior to dry immersion; D4 AM represents 4 days of dry immersion (morning prior to donning cuffs for the day for the cuff gr). D4 PM represent the data in the afternoon of day 4, after 10 h with cuff on, for the cuff gr.

Middle cerebral artery mean velocity was significantly lower at D4 AM compared to Pre (mean −16%, *p* = 0.0416) due to a drop in diastolic flow. Flows volume in the common carotid and superficial femoral arteries were unchanged after 4 days in dry immersion (all *p* > 0.05, Fig. 3). The flow redistribution ratio (carotid flow/femoral flow) did not change significantly.

### Cardiac and vascular data at D4 PM compared to D4 AM

The second hypothesis was to test the effect of wearing the cuff during the day and this test was done on Day 4 of the immersion by comparing morning (before donning cuffs: D4 AM) to afternoon (after having worn the cuffs for 8 h). However, when we consider the two groups separately, there is no difference between them nor at D4 AM nor D4 PM. Only the SV tended to increase in 12 of the 18 subjects (*p* < 0.09); none of the other parameters mentioned above showed significant change.

## Discussion

By the fourth day in the morning (D4 AM), the dry-immersion participants showed significant decreases in the left ventricle volume and stroke volume (approximately −14%, *p* < 0.05), which corresponded to the drop of ~15–20% in plasma volume^13,14^. It has been shown previously that DI, along with head-down tilt (HDT) bed rest, induces hypovolemia via a neuro-hormonal response mediated by the carotid and cardiopulmonary baroreceptors^15–19^. It follows that the left ventricle filling pressure would be reduced, explaining the significant reduction in ejection fraction observed. It seems unlikely that this is due to a decrease in contractility, as there has been very little time for remodeling of the cardiac myocytes.

At D4 AM, no difference in LVDV, SV, and left ventricle mass was found between treatment groups, implying no memory effect of thigh cuffs on those variables. These cardiac parameters reset overnight. Nevertheless, there is some (albeit rather weak) evidence of a daytime effect on cardiopulmonary function: during the daytime period (8:00 am to 6:00 pm), the stroke volume tended to increase in 12 of the 18 subjects (*p* < 0.09), 6 in the control (no thigh cuffs) and 6 in the thigh-cuff group. During previous HDBR and spaceflight experiments, the decreased left ventricle volume was restored to pre-bed rest/pre-flight values after 8 h wearing thigh cuffs. This is in contradiction to the present DI study where no significant change was found with cuff intervention^11,12^, suggesting that constant hydrostatic compression created by immersion actively counteracts and hides intermittent sequestrating effect of cuffs.

These trends to decrease observed for the cardiac volume are probably related, again, to the aforementioned hypovolemia, reduced preload, and deconditioning; there is no exercise at all while in the tank.

The interesting observations of this study are, instead, related to neck level (carotid stiffness) changes and leg level (femoral wall thickness: IMT). Common carotid artery distensibility index decreased significantly, suggesting increased stiffness in the vessel wall. This distensibility measure is conservative, because it assumes no change in carotid distending pressure from Pre to Day 4AM and PM. In spaceflight, carotid distending pressure would have been expected to increase compared to pre-flight (standing) due to fluidshift-induced pressure redistribution; however, in the DI model the hydrostatic pressure remains unchanged at the neck level. The scans were done in the same semi-recumbent position between Pre and Day 4AM and PM. Thus, to see a change in distensibility on the fourth day, although assuming no change in carotid distending pressure, suggests that even 4 days of DI is sufficient to induce arterial stiffness increase. Nevertheless, the change in distensibility were far below the magnitude of the significant change observed during and right after long-duration spaceflight (increased in and post flight ~15%)^6,7^, indicating that the 4-day dry-immersion stimulus was not sufficient to reproduce the published effects observed with 6-month spaceflight. There was no cumulate or “memory” effect of wearing cuffs during the day for 3 days in DI on the carotid stiffness. There was also no acute effect after 8 h with cuff on.

The carotid to tibial pulse wave velocity tended to increase in 10/18 subjects (*p* = 0.07), which is in favor of an increased stiffness of the distal leg arteries. It is interesting to consider this tendency in parallel with the increase in surrounding muscle stiffness (Tibial lateralis) as reported during a 7-day immersion^20^. Nevertheless, it is not possible from these to make a relationship between changes in muscle stiffness and arterial stiffness, further work is required.

Although the carotid stiffness increased significantly, the carotid IMT increased in 4/18 subject only (20%), contrary to what observed during spaceflight and confinement (~75% of the subjects)^6^. Carotid stiffness and IMT has been shown to not be correlated in space studies^6^, meaning that the mechanisms underlying these two processes are likely different and this is confirmed by DI.

Nevertheless, we did expect to see increases in carotid to mirror the observed femoral IMT increase. This speaks to our concern that the stimulus was insufficient to gain significance but perhaps sufficient to start the physiological process in some participants. Given a stronger DI stimulus (i.e., more time in the tank and deeper body immersion for higher hydrostatic pressure on the body), it seems logical to expect that the carotid IMT would likely have followed the femoral one. For IMT to change, cellular remodeling must have occurred, which appears to require more than 4 days in dry immersion to get underway in the carotids of most participants. It is helpful to keep in mind that microgravity alone cannot explain why the carotid and femoral IMT changes as increased of this parameter is also observed in Earth-based confinement. Intima media remodeling, then, appears to have more to do with hypoactivity, nutrition, and stress than with any pressure or flow redistribution that microgravity and dry immersion will induce.

Carotid and femoral flows (ml/min) did not change after 4 days in immersion, nor did the carotid to femoral flow ratio. This was expected, because previous dry-immersion studies showed that 2–3 h in the tank induces notable fluid shifts towards the head and thorax with the internal jugular vein significantly enlarged and the intracranial pressure increased in 50% of the subjects, but the carotid flow remains stable even after this period when the subject is already becoming hypovolemic^2^. The cuffs did not affect arterial femoral flow, because they are of low pressure and only superficial leg veins were compressed. The venous return through the deep veins remains unchanged and therefore the arterial flow proceeded unaffected.

Conversely, the mean flow velocity in the middle cerebral artery was reduced with dry immersion (12/18 subjects), which means that, barring any diameter change, the flow volume in this artery should also have been reduced (around 15%) in these subjects. This may be related to the reduction in diastolic flow in relation with the drop in circulating blood flow and stroke volume. Finally, the cerebral flow was slightly reduced at Day 4 in the morning in all subjects, which is in agreement with the decrease in intracranial arterial linear velocity already reported during a 7-day DI by Moreva et al.^21^. The mean cerebral arterial velocity showed a tendency to increase during daytime (D4 AM to PM) in both groups concomitantly with the increase in stroke volume during the same period, probably in relation to daytime activity. Again, the thigh cuffs had no significant effect on the arterial cerebral flow.

During short-duration HDBR or short-duration confinement (4 days), the carotid and femoral vascular resistance and flow also remained unchanged or showed very limited variation, similar to the current study of the same duration, whereas the arterial response to orthostatic test after the 4 days was, however, affected^22^. This reinforces the idea that 4 days of either dry-immersion, confinement, or bed rest is not long enough to induce remodeling and significant hemodynamic changes at rest, but can still have an impact on arterial dynamic function such as active vasoconstriction or dilatory capacity in response to acute fluid transfer (mediated by tilt or LBNP for example).

The thigh-cuff countermeasure was proposed for the current study, because it was found to have a significant beneficial impact inflight on the cervical venous engorgement and head stuffiness, the stroke volume, and the arterial vasomotor activity during orthostatic tests. In the present experiment, the thigh cuffs were worn for 8 h per day and had a limited impact on the arterial network. At Day 4 in the morning before donning the cuffs, none of the arterial parameters presented above showed a cumulative cuff effect; the two groups were indistinguishable in the morning. Moreover, the trends and tendencies observed in some parameters were often equally distributed between the two groups, adding to the explanation that the countermeasure had limited impact on the arterial network.

Previously, the same cuff countermeasure was tested during a 7-day bed rest and 6-month spaceflights, and again, no effect was detected on the arterial hemodynamics (specifically carotid and femoral flows) and no “cuff memory” effect was found at day 7 in the morning after a night without cuffs, or at the end of the spaceflight^11,12^. This is consistent with our previous observations after 4 days in dry immersion^2^. Nevertheless, in bed rest or spaceflight, the thigh-cuff countermeasure induced more change after 8 h compared to that observed after 8 h with cuff in dry immersion. This might be explained by the fact that in HDBR the fluid transfer towards the head induced by the head-down tilt position is facilitated by the gravity vector from feet to head and during spaceflight by the suppression of gravity, whereas in dry immersion the gravity vector is opposite and counteracts the cephalad fluidshift centralizing effect of immersion hides sequestrating effect of cuffs.

In a previous study^2^, we reported that the dry immersion induces a significant headwards fluidshift (similar to in space) with a significant increase in jugular volume and intracranial vein velocity but only during the first 3 h. After 1 day, the important drop in plasma volume and the persistence of a vertical downward gravity vector reduced partially the fluidshift effect at the head level. Thus, dry immersion mimics quite well the microgravity induced fluidshift but during a limited number of hours.

On the arterial side, the present 4-day duration of the dry immersion was not sufficient for inducing significant vessel wall remodeling such as that already observed during long-duration HDT, spaceflight, or confinement.

Lastly, the position of the subjects inside the bath is not easy to control, as they can put their legs close to the water surface, which reduce the pressure applied to the legs and thus the dry-immersion effect. Dry immersion induced a cardiac volume decrease without evidence of contractility decrease. Dry immersion also induced a decrease in arterial distensibility and large artery IMT changes, at least in half the population, both at the carotid and femoral level, whereas the corresponding peripheral flows remained unchanged. Dry immersion induced the same type of cardiac and arterial changes as observed during spaceflight (and with age on earth); however, this method of simulation of microgravity effect must be applied for a longer period than 4 days time to achieve the appropriate and comparable dose effect. In this way, DI should be sufficient to test countermeasure such as thigh cuffs.

## Methods

### Subjects

A total of 18 subjects were included in the study and randomly divided at DI-2 days into Control (Co) or Cuffs group (CU) (nine/nine split). All subjects were informed about the experimental procedures and gave their written informed consent. The experimental protocol conformed to the standards set by the Declaration of Helsinki and was approved by the local Ethics Committee (CPP Est III: 2 October 2018, number ID RCB 2018-A01470-55) and French Health Authorities (ANSM: 13 August 2018). Clinical Trials.gov Identifier:NCT03915457. There was no significant difference in any of group characteristics between groups at baseline (Table 1).

The study was conducted at the MEDES Space Clinic, Toulouse, France. The experimental protocol included 4 days of ambulatory baseline measurements before immersion (DI-4 to DI-1), 5 days (120 h) of dry immersion (DI-1 to DI-5), and 2 days of ambulatory recovery (R0, R + 1). Subjects randomized to Cuffs group wore the thigh cuffs during the 5 days of DI, from 10 to 18 h at DI-1 and from 8 to 18 h at DI-2 to DI-5. Thigh cuffs are elastic straps customized to each subject, to have the same effects on lower-limb distensibility as at pressure level of ~30 mm Hg. Individual adjustment was determined for each subject with calf plethysmography, performed in the supine position at DI-2. General protocol of strict DI was conducted according to methodology detailed in De Abreu et al.^23^. Thermo-neutral water temperature was continuously maintained. Light-off period was set at 23:00–07:00 h. Daily hygiene, weighing, and some specific measurements required extraction from the bath. During these out-of-bath periods, subjects maintained the −6° head-down position. On DI-1 to DI-4, out-of-bath time was 1.1 ± 0.6 h/day. Otherwise, during DI, subjects remained immersed in a supine position for all activities and were continuously observed by video monitoring. Body weight, blood pressure, heart rate, and tympanic body temperature were measured daily. The frames of adequate water intake were fixed at 35–60 ml/kg/day; within these frames, water intake throughout the protocol was ad libitum (measured). The menu composition of each experiment day was identical for all participants and dietary intake was individually tailored and controlled during the study.

### Experimental protocol

Participants were assigned into either the control or cuffs group (nine/nine split). The cuffs group wore the Russian “Braslets” device (“cuffs”)^24^ around both upper thighs tightened to 30 mm Hg. Calibration was performed using plethysmography combined with direct ultrasound measurements of the popliteal vein diameter. Cuffs were worn for ten consecutive daytime hours only, while immersed to the neck in the tank. The control group wore no cuffs while in the tank.

Ultrasound measurements were taken (Orcheo-Lite, Sonoscanner, Paris, France) 3 days before entering the tank (Pre) in a semi-recumbent position to simulate the DI posture without immersion. As the fifth day was very busy with several measures requiring period outside the tank (biopsy, computed tomography, magnetic resonance imaging), our last measure occurred on the evening of day 4. Measurements were repeated again twice more on the fourth day in the tank: in the morning (D4 AM) prior to donning the cuffs for the day (cuffs group) and again in the afternoon (D4 PM), with cuffs in place, after having worn the cuffs for 8 h (cuffs group).

Participants were passively lifted from the tank once per day in the morning for showering and toileting, while remaining supine during their entire time out of the tank. As other experiments required additional out-of-tank time, the total out-of-bath time was kept to ~1 h per day (or less) between days 1 and 4. Participants were not permitted caffeine, alcohol, or any strenuous physical activity for 24 h prior to the ultrasound sessions. There was no physical exercise in the tank.

### Parameters measured

- Diameters were measured manually from the B-mode images using calipers placed by the same trained sonographer, while IMT measurements were semi-automated using the ultrasound radiofrequency signal (RF) processing (Sonoscanner software Version 8.4.101.99).
- Propagation wave velocity (PWV) was measured using the time between the ECG R wave and the Doppler peak systole.
- Flow volumes in the main arteries were measured as follows:
  Common carotid: Qcc (ml/min) = mean velocity × vessel sectional area
  Superficial femoral artery Qfem = mean velocity × vessel sectional area
  Middle cerebral artery flow Fmca = mean velocity (due to vessel section not being measurable)^25^
  Flow redistribution ratio (carotid flow/femoral flow) = Qcc/Qfem
- Distensibility Index was calculated using the following formula:^6^ DI = (Dd − Ds)/Ds, where Dd = arterial diameter at end diastole and Ds = arterial diameter at end systole
- Cardiac volume and mass was calculated: left ventricle diastolic volume: LVDV (cm^3^) = [(7 × LVDD^3^)/(2,4 + LVDD)] with LVDD and LVSD left ventricle diastolic and systolic diameter^26^.

Left ventricle mass: LVmass(g) = 0.80 [1.04{(PWT + LVDD + SWT)^3^ − LVID^3^ }] + 0.6

SWT = septal wall thickness, PWT = posterior wall thickness^27^.

### Statistical analysis

To test the hypothesis that wearing the cuffs for 10 h during dry immersion affected cardiac function, arterial wall properties and arterial blood flow properties, a dependent *t*-test was used to compare D4 AM to D4 PM, preceded by a Shapiro test for normality. To test the hypothesis that 4 days of dry immersion affected these main outcomes, a 2 × 2 repeated-measures analysis of variance was used to compare Pre to D4 AM with a main effects analysis for time (DI effect) and condition (Cuff effect), followed by a Shapiro test for normality on the residuals. Significance was set at *P* < 0.05; values are reported as mean ± SD. All tests were coded in R 3.6.0 (R Core Team, The Netherlands).

### Reporting summary

Further information on research design is available in the Nature Research Reporting Summary linked to this article.

## Supplementary information

Reporting Summary

## Data Availability

The corresponding author will be responsible for materials availability. There is no restriction on data availability.
