# Supplementary material for: 4 Day in dry immersion reproduces partially the aging effect on the arteries as observed during 6 month spaceflight or confinement
Source: NPJ Microgravity. 2021 Nov 2;7:43. doi: 10.1038/s41526-021-00172-6 (PMC8564509; doi:10.1038/s41526-021-00172-6)
Supplement: Supplementary file 1 — Reporting Summary [file 41526_2021_172_MOESM1_ESM.pdf]

nature portfolio

Double-blind peer review submissions: write
Corresponding author(s): Philippe ARBEILLE
Last updated by author(s): August 23th 2021

Reporting Summary

Nature Portfolio wishes to improve the reproducibility of the work that we publish. This form provides structure for consistency and transparency in reporting. For further information on Nature Portfolio policies, see our Editorial Policies and the Editorial Policy Checklist.

Statistics

For all statistical analyses, confirm that the following items are present in the figure legend, table legend, main text, or Methods section.

n/a	Confirmed	
	X	The exact sample size (n) for each experimental group/condition, given as a discrete number and unit of measurement
X		A statement on whether measurements were taken from distinct samples or whether the same sample was measured repeatedly
	X	The statistical test(s) used AND whether they are one- or two-sided<br>*Only common tests should be described solely by name; describe more complex techniques in the Methods section.*
	X	A description of all covariates tested
	X	A description of any assumptions or corrections, such as tests of normality and adjustment for multiple comparisons
	X	A full description of the statistical parameters including central tendency (e.g. means) or other basic estimates (e.g. regression coefficient) AND variation (e.g. standard deviation) or associated estimates of uncertainty (e.g. confidence intervals)
	X	For null hypothesis testing, the test statistic (e.g. F, t, r) with confidence intervals, effect sizes, degrees of freedom and P value noted<br>*Give P values as exact values whenever suitable.*
X		For Bayesian analysis, information on the choice of priors and Markov chain Monte Carlo settings
X		For hierarchical and complex designs, identification of the appropriate level for tests and full reporting of outcomes
	X	Estimates of effect sizes (e.g. Cohen's d, Pearson's r), indicating how they were calculated

Our web collection on statistics for biologists contains articles on many of the points above.

Software and code

Policy information about availability of computer code

Data collection: No software was used to collect the data (measures performed directly on the Echograph).

Data analysis: t-test, Shapiro test, ANOVA, All tests were coded in R 3.6.0 (R Core Team, Netherlands)

For manuscripts utilizing custom algorithms or software that are central to the research but not yet described in published literature, software must be made available to editors and reviewers. We strongly encourage code deposition in a community repository (e.g. GitHub). See the Nature Portfolio guidelines for submitting code & software for further information.

Data

Policy information about availability of data

All manuscripts must include a data availability statement. This statement should provide the following information, where applicable:

- Accession codes, unique identifiers, or web links for publicly available datasets
- A description of any restrictions on data availability
- For clinical datasets or third party data, please ensure that the statement adheres to our policy

The corresponding author will be responsible for materials availability. There is no restriction on data availability.

Field-specific reporting

Please select the one below that is the best fit for your research. If you are not sure, read the appropriate sections before making your selection.

[X] Life sciences [ ] Behavioural & social sciences [ ] Ecological, evolutionary & environmental sciences

For a reference copy of the document with all sections, see nature.com/documents/nr-reporting-summary-flat.pdf

Life sciences study design

All studies must disclose on these points even when the disclosure is negative.

Sample size	18 subjects in dry immersion (9 countermeasure & 9 control)
Data exclusions	exclusively males. History of cardiovascular or other chronic diseases. Presence of acute or chronic pathologies, which could affect the physiological data. Smokers. Taking medication or drugs prior to the experiment. Normal clinical and paramedical examination and laboratory tests (hematology and blood chemistry).
Replication	No replication
Randomization	Allocation of the subject in the 2 groups randomized
Blinding	Blinding not possible as the sonographer was performing the ultrasound images directly on the subject

Behavioural & social sciences study design

All studies must disclose on these points even when the disclosure is negative.

Study description	Allocation of the 18 subject in the 2 groups
Research sample	18 volunteers in 2groups (countermeasure 9 – control 9)
Sampling strategy	The Dry immersion experiment was designed by CNES (drench space agency) and MEDES (institute for spave medicine) . They considered that 9 subjects per group will be sufficient for statistical analysis. Moreover there is only 2 dry immersion bath which do not allow to investigate easily a higher number of subject.
Data collection	Ultrasound data collected by a sonographer directly on the subject
Timing	Ultrasound data collected pre dry immersion and at Day4 AM and PM
Data exclusions	exclusively males. History of cardiovascular or other chronic diseases. Presence of acute or chronic pathologies, which could affect the physiological data. Smokers. Taking medication or drugs prior to the experiment. Normal clinical and paramedical examination and laboratory tests (hematology and blood chemistry).
Non-participation	No dropped out
Randomization	Allocation of subjects into the 2 groups randomized

Ecological, evolutionary & environmental sciences study design

All studies must disclose on these points even when the disclosure is negative.

Study description	NA: Non applicable
Research sample	NA: Non applicable

Research sample | NA], and provide a rationale for the sample choice. When relevant, describe the organism taxa, source, sex, age range and ...lations. State what population the sample is meant to represent when applicable. For studies involving existing datasets, ...e data and its source.

Sampling strategy | NA ...mpling procedure. Describe the statistical methods that were used to predetermine sample size OR if no sample-size ... was performed, describe how sample sizes were chosen and provide a rationale for why these sample sizes are sufficient.

Data collection | NA ...e data collection procedure, including who recorded the data and how.

Timing and spatial scale | NA ...start and stop dates of data collection, noting the frequency and periodicity of sampling and providing a rationale for ...es. If there is a gap between collection periods, state the dates for each sample cohort. Specify the spatial scale from which the data are taken

Data exclusions | NA ...were excluded from the analyses, state so OR if data were excluded, describe the exclusions and the rationale behind them, ...whether exclusion criteria were pre-established.

Reproducibility | NA ...e measures taken to verify the reproducibility of experimental findings. For each experiment, note whether any attempts to ... experiment failed OR state that all attempts to repeat the experiment were successful.

Randomization | NA ...allocation was not random, describe how covariates were

Blinding | Describe the extent of blinding used during data acquisition and analysis. If blinding was not possible, describe why OR explain why blinding was not relevant to your study.

Did the study involve field work? ☐ Yes X No

Field work, collection and transport

Field conditions | NA ...e study conditions for field work, providing relevant parameters (e.g. temperature, rainfall).

Location | NA ...ation of the sampling or experiment, providing relevant parameters (e.g. latitude and longitude, elevation, water depth).

Access & import/export | NA ...e efforts you have made to access habitats and to collect and import/export your samples in a responsible manner and in ...with local, national and international laws, noting any permits that were obtained (give the name of the issuing authority, the date of issue, and any identifying information).

Disturbance | NA ...ny disturbance caused by the study and how it was minimized.

Reporting for specific materials, systems and methods

We require information from authors about some types of materials, experimental systems and methods used in many studies. Here, indicate whether each material, system or method listed is relevant to your study. If you are not sure if a list item applies to your research, read the appropriate section before selecting a response.

Materials & experimental systems

n/a	Involved in the study
X	☐ Antibodies
X	☐ Eukaryotic cell lines
X	☐ Palaeontology and archaeology
X	☐ Animals and other organisms
☐	X Human research participants
X	☐ Clinical data
X	☐ Dual use research of concern

Methods

n/a	Involved in the study
X	☐ ChIP-seq
X	☐ Flow cytometry
X	☐ MRI-based neuroimaging

Antibodies

Antibodies used | NA ...l antibodies used in the study; as applicable, provide supplier name, catalog number, clone name, and lot number.

Validation | NA ...e validation of each primary antibody for the species and application, noting any validation statements on the ...rer's website, relevant citations, antibody profiles in online databases, or data provided in the manuscript.

Eukaryotic cell lines

Policy information about cell lines

Cell line source(s) | NA ...ource of each cell line used.

Authentication	NA	...e authentication procedures for each cell line used OR declare that none of the cell lines used were authenticated.
Mycoplasma contamination	NA	...t all cell lines tested negative for mycoplasma contamination OR describe the results of the testing for ...a contamination OR declare that the cell lines were not tested for mycoplasma contamination.
Commonly misidentified lines (See ICLAC register)	NA	...commonly misidentified cell lines used in the study and provide a rationale for their use.

Palaeontology and Archaeology

Specimen provenance	NA	...venance information for specimens and describe permits that were obtained for the work (including the name of the ...hority, the date of issue, and any identifying information). Permits should encompass collection and, where applicable, export.
Specimen deposition	NA	...here the specimens have been deposited to permit free access by other researchers.
Dating methods	NA	...s are provided, describe how they were obtained (e.g. collection, storage, sample pretreatment and measurement), where ...btained (i.e. lab name), the calibration program and the protocol for quality assurance OR state that no new dates are provided.

☐ Tick this box to confirm that the raw and calibrated dates are available in the paper or in Supplementary Information.

Ethics oversight	NA	...organization(s) that approved or provided guidance on the study protocol, OR state that no ethical approval or guidance ...d and explain why not.

Note that full information on the approval of the study protocol must also be provided in the manuscript.

Animals and other organisms

Policy information about studies involving animals; ARRIVE guidelines recommended for reporting animal research

Laboratory animals	NA	...ry animals, report species, strain, sex and age OR state that the study did not involve laboratory animals.
Wild animals	NA	Provide details on animals observed in or captured in the field; report species, sex and age where possible. Describe how animals were ...transported and what happened to captive animals after the study (if killed, explain why and describe method; if released, ...and when) OR state that the study did not involve wild animals.
Field-collected samples	NA	...ry work with field-collected samples, describe all relevant parameters such as housing, maintenance, temperature, ...d and end-of-experiment protocol OR state that the study did not involve samples collected from the field.
Ethics oversight	NA	...organization(s) that approved or provided guidance on the study protocol, OR state that no ethical approval or guidance ...d and explain why not.

Note that full information on the approval of the study protocol must also be provided in the manuscript.

Human research participants

Policy information about studies involving human research participants

Population characteristics	Subjects were volunteers, Caucasian Males aged 33,9+/7y (control gr) 34,1+/-3,7y (countermeasure gr) and free from any pathology nor treatment.
Recruitment	Recruitment announcement on ESA/CNES site
Ethics oversight	The experimental protocol conformed to the standards set by the Declaration of Helsinki and was approved by the local Ethics Committee (CPP Est III: October 2, 2018, n° ID RCB 2018-A01470-55) and French Health Authorities (ANSM: August 13, 2018). Clinical Trials.gov Identifier: NCT03915457.

Note that full information on the appro...

Clinical data

Policy information about clinical studies

All manuscripts should comply with the ICMJE guidelines for publication of clinical research and a completed CONSORT checklist must be included with all submissions.

Clinical trial registration	NA	...trial registration number from ClinicalTrials.gov or an equivalent agency.
Study protocol	NA	...the full trial protocol can be accessed OR if not available, explain why.
Data collection	NA	...e settings and locales of data collection, noting the time periods of recruitment and data collection.
Outcomes	NA	...w you pre-defined primary and secondary outcome measures and how you assessed these measures.

Dual use research of concern

Policy information about dual use research of concern

Hazards

Could the accidental, deliberate or reckless misuse of agents or technologies generated in the work, or the application of information presented in the manuscript, pose a threat to:

No	Yes	
X	☐	Public health
X	☐	National security
X	☐	Crops and/or livestock
X	☐	Ecosystems
X	☐	Any other significant area

Experiments of concern

Does the work involve any of these experiments of concern:

No	Yes	
X	☐	Demonstrate how to render a vaccine ineffective
X	☐	Confer resistance to therapeutically useful antibiotics or antiviral agents
X	☐	Enhance the virulence of a pathogen or render a nonpathogen virulent
X	☐	Increase transmissibility of a pathogen
X	☐	Alter the host range of a pathogen
X	☐	Enable evasion of diagnostic/detection modalities
X	☐	Enable the weaponization of a biological agent or toxin
X	☐	Any other potentially harmful combination of experiments and agents

ChIP-seq

Data deposition

No :onfirm that both raw and final processed data have been deposited in a public database such as GEO.

No :onfirm that you have deposited or provided access to graph files (e.g. BED files) for the called peaks.

Data access links<br>*May remain private before publication.*	NO *[illegible] submission" or "Revised version" documents, provide reviewer access links. For your "Final submission" document, [illegible] link to the deposited data.*
Files in database submission	NO *list of all files available in the database submission.*
Genome browser session<br>(e.g. UCSC)	NO *link to an anonymized genome browser session for "Initial submission" and "Revised version" documents only, to [illegible] peer review. Write "no longer applicable" for "Final submission" documents.*

Methodology

Replicates	NA *the experimental replicates, specifying number, type and replicate agreement.*
Sequencing depth	NA *the sequencing depth for each experiment, providing the total number of reads, uniquely mapped reads, length of reads and they were paired- or single-end.*
Antibodies	NA *the antibodies used for the ChIP-seq experiments; as applicable, provide supplier name, catalog number, clone name, and lot*
Peak calling parameters	NA *e command line program and parameters used for read mapping and peak calling, including the ChIP, control and index files*
Data quality	NA *the methods used to ensure data quality in full detail, including how many peaks are at FDR 5% and above 5-fold enrichment.*
Software	NA *the software used to collect and analyze the ChIP-seq data. For custom code that has been deposited into a community [illegible], provide accession details.*

Flow Cytometry NA

Plots

Confirm that: NA

NA ie axis labels state the marker and fluorochrome used (e.g. CD4-FITC).

NA ie axis scales are clearly visible. Include numbers along axes only for bottom left plot of group (a 'group' is an analysis of identical markers).

NA ll plots are contour plots with outliers or pseudocolor plots.

NA numerical value for number of cells or percentage (with statistics) is provided.

Methodology

Sample preparation	*Describe the sample preparation, detailing the biological source of the cells and any tissue processing steps used.*
Instrument	*Identify the instrument used for data collection, specifying make and model number.*
Software	*Describe the software used to collect and analyze the flow cytometry data. For custom code that has been deposited into a community repository, provide accession details.*
Cell population abundance	*Describe the abundance of the relevant cell populations within post-sort fractions, providing details on the purity of the samples and how it was determined.*
Gating strategy	*Describe the gating strategy used for all relevant experiments, specifying the preliminary FSC/SSC gates of the starting cell population, indicating where boundaries between "positive" and "negative" staining cell populations are defined.*

☐ Tick this box to confirm that a figure exemplifying the gating strategy is provided in the Supplementary Information.

Magnetic resonance imaging NA

Experimental design NA

Design type	*Indicate task or resting state; event-related or block design.*
Design specifications	*Specify the number of blocks, trials or experimental units per session and/or subject, and specify the length of each trial or block (if trials are blocked) and interval between trials.*
Behavioral performance measures	*State number and/or type of variables recorded (e.g. correct button press, response time) and what statistics were used to establish that the subjects were performing the task as expected (e.g. mean, range, and/or standard deviation across subjects).*

Acquisition NA

Imaging type(s)	*Specify: functional, structural, diffusion, perfusion.*
Field strength	*Specify in Tesla*
Sequence & imaging parameters	*Specify the pulse sequence type (gradient echo, spin echo, etc.), imaging type (EPI, spiral, etc.), field of view, matrix size, slice thickness, orientation and TE/TR/flip angle.*
Area of acquisition	*State whether a whole brain scan was used OR define the area of acquisition, describing how the region was determined.*

Diffusion MRI ☐ Used ☐ Not used

Preprocessing NA

Preprocessing software	*Provide detail on software version and revision number and on specific parameters (model/functions, brain extraction, segmentation, smoothing kernel size, etc.).*
Normalization	*If data were normalized/standardized, describe the approach(es): specify linear or non-linear and define image types used for transformation OR indicate that data were not normalized and explain rationale for lack of normalization.*
Normalization template	*Describe the template used for normalization/transformation, specifying subject space or group standardized space (e.g. original Talairach, MNI305, ICBM152) OR indicate that the data were not normalized.*
Noise and artifact removal	*Describe your procedure(s) for artifact and structured noise removal, specifying motion parameters, tissue signals and physiological signals (heart rate, respiration).*

Volume censoring | Define your software and/or method and criteria for volume censoring, and state the extent of such censoring.

Statistical modeling & inference NA

Model type and settings | Specify type (mass univariate, multivariate, RSA, predictive, etc.) and describe essential details of the model at the first and second levels (e.g. fixed, random or mixed effects; drift or auto-correlation).

Effect(s) tested | Define precise effect in terms of the task or stimulus conditions instead of psychological concepts and indicate whether ANOVA or factorial designs were used.

Specify type of analysis: ☐ Whole brain ☐ ROI-based ☐ Both

Statistic type for inference (See Eklund et al. 2016) | Specify voxel-wise or cluster-wise and report all relevant parameters for cluster-wise methods.

Correction | Describe the type of correction and how it is obtained for multiple comparisons (e.g. FWE, FDR, permutation or Monte Carlo).

Models & analysis NA

n/a	Involved in the study
☐	☐ Functional and/or effective connectivity
☐	☐ Graph analysis
☐	☐ Multivariate modeling or predictive analysis

Functional and/or effective connectivity | Report the measures of dependence used and the model details (e.g. Pearson correlation, partial correlation, mutual information).

Graph analysis | Report the dependent variable and connectivity measure, specifying weighted graph or binarized graph, subject- or group-level, and the global and/or node summaries used (e.g. clustering coefficient, efficiency, etc.).

Multivariate modeling and predictive analysis | Specify independent variables, features extraction and dimension reduction, model, training and evaluation metrics.
